# Functional Profiling Reveals Altered Metabolic Activity in Divers’ Oral Microbiota During Commercial Heliox Saturation Diving

**DOI:** 10.3389/fphys.2021.702634

**Published:** 2021-10-13

**Authors:** Roxane Monnoyer, Ingrid Eftedal, Astrid Hjelde, Sanjoy Deb, Kjersti Haugum, Jacky Lautridou

**Affiliations:** ^1^Department of Circulation and Medical Imaging, Faculty of Medicine and Health Sciences, Norwegian University of Science and Technology, Trondheim, Norway; ^2^Faculty of Nursing and Health Sciences, Nord University, Bodø, Norway; ^3^Centre for Nutraceuticals, School of Life Sciences, University of Westminster, London, United Kingdom; ^4^Department of Clinical and Molecular Medicine, Faculty of Medicine and Health Sciences, Norwegian University of Science and Technology, Trondheim, Norway; ^5^Department of Medical Microbiology, Clinic of Laboratory Medicine, St. Olavs Hospital, Trondheim University Hospital, Trondheim, Norway

**Keywords:** 16S rRNA sequencing, hyperbaric stress, hyperbaric hyperoxia, oral microbiome, oxidative stress, vitamin B_12_, energy metabolism

## Abstract

**Background:** The extreme environment in saturation diving affects all life forms, including the bacteria that reside on human skin and mucosa. The oral cavity alone is home to hundreds of different bacteria. In this study, we examined the metabolic activity of oral bacteria from healthy males during commercial heliox saturation diving. We focused on environmentally induced changes that might affect the divers’ health and fitness.

**Methods:** We performed pathway abundance analysis using PICRUSt2, a bioinformatics software package that uses marker gene data to compute the metabolic activity of microbial communities. The analysis is based on 16S rRNA metagenomic data generated from the oral microbiota of 23 male divers before, during, and after 4weeks of commercial heliox saturation diving. Environmentally induced changes in bacterial metabolism were computed from differences in predicted pathway abundances at baseline before, versus during, and immediately after saturation diving.

**Results and Conclusion:** The analysis predicted transient changes that were primarily associated with the survival and growth of bacteria in oxygenated environments. There was a relative increase in the abundance of aerobic metabolic pathways and a concomitant decrease in anaerobic metabolic pathways, primarily comprising of energy metabolism, oxidative stress responses, and adenosylcobalamin biosynthesis. Adenosylcobalamin is a bioactive form of vitamin B_12_ (vitB_12_), and a reduction in vitB_12_ biosynthesis may hypothetically affect the divers’ physiology. While host effects of oral bacterial vitamin metabolism are uncertain, this is a finding that concurs with the existing recommendations for vitB_12_ supplements as part of the divers’ diet, whether to boost antioxidant defenses in bacteria or their host or to improve oxygen transport during saturation diving.

## Introduction

During commercial saturation diving, divers may reside for weeks in a pressurized environment while breathing a mix of oxygen and helium (heliox). They must acclimatize to the extreme environment, including elevated partial pressures of oxygen (ppO_2_). Hyperoxia is a powerful driver of biological processes (Kiboub et al., 2018a,b; Luczynski et al., 2019). In saturation diving, the divers are exposed to hyperbaric hyperoxia, and oxygen also affects the composition of the microbiota: the microbial communities that reside on human skin and mucosa (Loesche et al., 1983; Rivera-Chávez et al., 2017). These microbiota have co-evolved with their human hosts and contribute symbiotically to maintain ecological and physiological balance (Cornejo Ulloa et al., 2019).

More than 700 bacterial species have been detected in the human oral cavity, with any one single individual’s mouth harboring up to 200 distinct bacteria (Dewhirst et al., 2010). This makes it the second most complex of the human microbiota after that of the gut (Kilian et al., 2016). The constant contact between the oral cavity and the external environment contributes to a large inter-individual diversity in microbial composition (Griffen et al., 2012; Sato et al., 2015). While they are primarily recognized for their role in digestion and oral diseases, oral bacteria can also modulate human physiology and pathology (Sampaio-Maia et al., 2016). Commercial divers are generally healthy and must satisfy specific requirements for fitness but it is unknown whether and how environmentally induced changes to their oral microbiota may challenge their physiology.

We have previously reported a transient shift in the composition of the oral microbiota during commercial saturation diving (Monnoyer et al., 2021). The shift reflected changes in oxygen availability and consisted primarily of an increase in aerobic and oxygen tolerant bacteria at the expense of obligate anaerobes. In the current study, we aimed to predict environmentally induced changes in bacterial metabolism. We used metagenomic data to model metabolic pathways and processes before, during, and after saturation diving, hypothesizing that there would be a relative increase in oxidative respiration during the bottom phase and a concomitant decrease in anaerobic fermentation. The resultant predictions may be examined as targets for modulation through interventions such as nutrition.

## Materials and Methods

### Ethics

This study was conducted on metagenome data from the oral microbiota of divers who participated in a commercial diving campaign in the Mediterranean Sea in 2018. The protocol was approved in advance by the Norwegian Regional Committee for Medical and Health Research Ethics (REK), number 2018/1184. The participants were informed of the aims and scope of the study and their right to withdraw without repercussions and provided signed consent prior to inclusion. All experimental procedures were conducted according to the Declaration of Helsinki principles for ethical human experimentation. The divers’ characteristics and criteria for eligibility are presented in Monnoyer et al. (2021).

### Study Material

**Figure 1:**
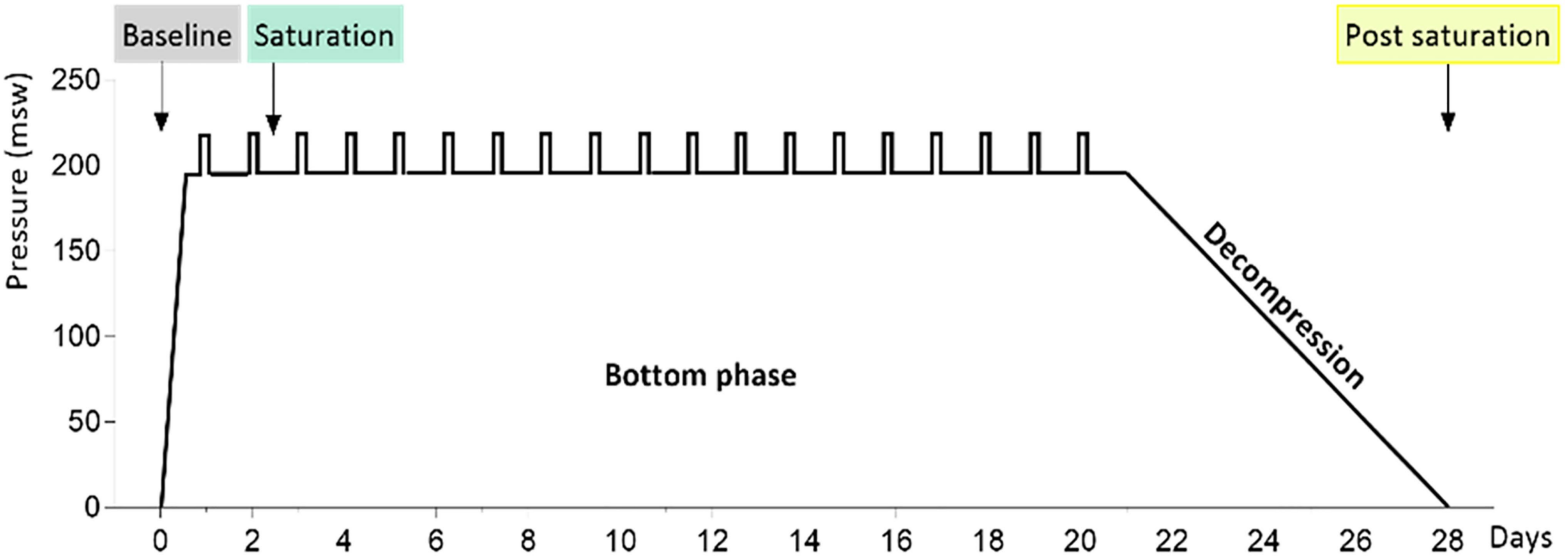
The 4-week hyperbaric saturation profile. Pressure is shown as meters of sea water (msw). The saturation was done in a heliox atmosphere, with 6-h compression, app. 21-day bottom phase at or near 200msw, and app. 8-day decompression. Daily bell runs are indicated by vertical bars on the bottom phase profile. Colored labels point to saliva collection times before, during, and after the saturation.

The study material was generated from saliva samples from 23 divers before, during, and after a 28-day saturation dive. The diving protocol has been presented previously by Luczynski et al. (2019). In brief, the divers were compressed over 6h to 182–200meters of sea water (msw). During the bottom phase, they worked in teams of three, with shifts lasting 12h. Each team did one underwater work excursion (bell run) per day to 192–210m, 7days per week. After the bottom phase, the divers were decompressed to atmospheric pressure over 8days. During the bottom phase, the ppO_2_ was kept at 40kPa in the living chamber and 60–80kPa during work. During the decompression, ppO_2_ was increased to 50kPa until 13msw and then gradually reduced to 21kPa until surface. Saliva samples were collected into OMNIgene ORAL OM-505-tubes (DNA Genotek, Ottawa, Canada) and sequenced on a Illumina NextSeq flowcell generating 16S rRNA metagenomic data, as described earlier (Monnoyer et al., 2021). The saturation diving profile and sample collection time points for this study are shown in Figure 1. The 16S rRNA fastq files are available at the European Nucleotide Archive (ENA) at EMBL-EBI under accession number PRJEB40804.^1^

### Bioinformatics

The fastq files were quality checked and trimmed before subjected to denoising by DADA2 (Callahan et al., 2016) to generate a table of Amplicon Sequence Variants (ASVs) counts for each sample. The taxonomic identity of each ASV was determined by region-specific classifiers trained on the SILVA database (v132; Quast et al., 2013). The ASV count table and taxonomic classifications were subjected to pathway abundance analysis by Phylogenetic Investigation of Communities by Reconstruction of Unobserved States (PICRUSt2; Douglas et al., 2020). Quality filters, denoising, taxonomic classification, and pathway abundance results were all generated using the Quantitative Insights Into Microbial Ecology (QIIME2, version 2019.10) framework (Bolyen et al., 2019). Significant differentially activated pathways were identified by paired *t* tests as implemented in the voom method in the limma R package (Law et al., 2014; Ritchie et al., 2015) using a Benjamini-Hochberg correction adjusted value of *p*<0.05. Outputs from the voom method can be found in Supplementary Data. The different time points were compared to the reference value using the individual dives as a blocking factor. Pathway’s abundances were summarized using the MetaCyc Metabolic Pathway database: https://metacyc.org/ (Caspi et al., 2020).

## Results

**Figure 2:**
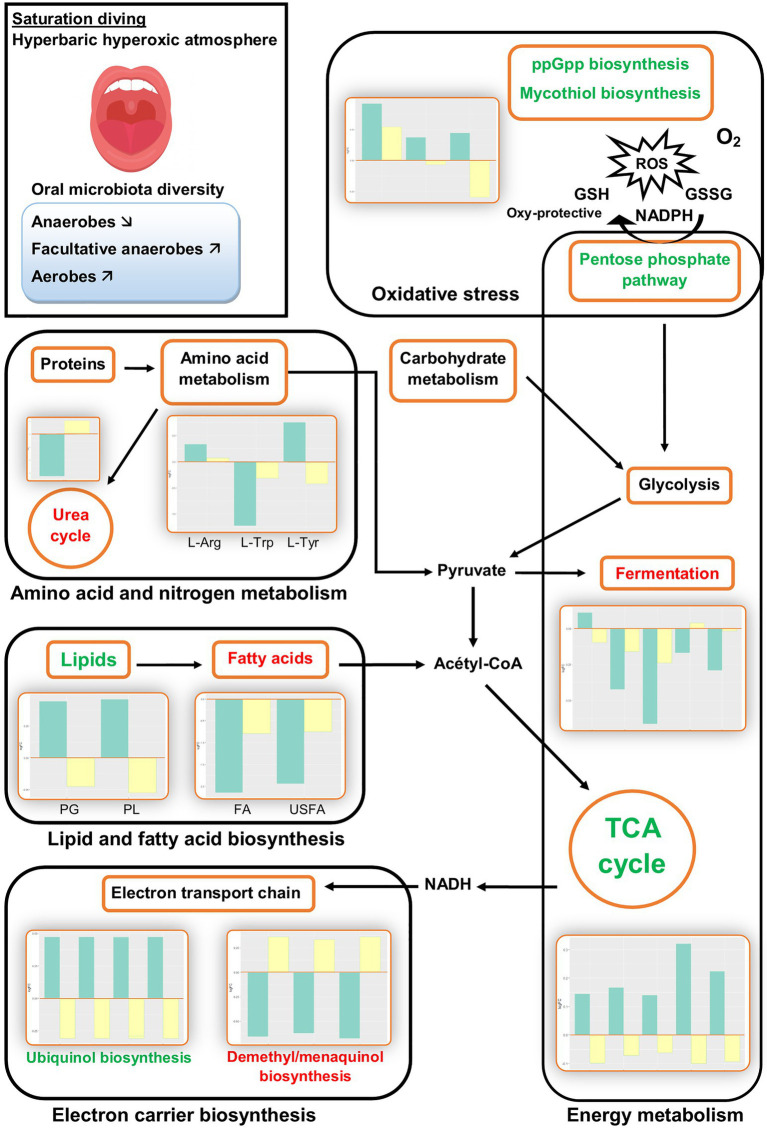
Illustrative overview of inferred changes in oral bacterial metabolic pathways during hyperbaric heliox saturation. The bar plots show log2 fold change from paired *t* tests (means relative to baseline, *n*=23): green=after 2days of heliox saturation; yellow=after decompression. For each pathway, the overall direction of change is indicated by green (more active) or red (less active) text. L-Arg, L-arginine biosynthesis (*p* = 0.0205); L-Trp, L-tryptophane degradation (*p* = 0.0066); L-Tyr, L-tyrosine degradation (*p* = 0.0337); PG, phosphatidylglycerol (*p*=0.0066); PL, phospholipid (*p*=0.0066); FA, fatty acids (*p*=0.0233); and USFA, unsaturated fatty acids (*p*=0.0209).

The PICRUSt2 analysis predicted several changes in biological pathways and processes during commercial saturation diving. All changes were transient: They were observed only during the bottom phase and disappeared after the decompression. Figure 2 summarizes the main results during the bottom phase (green bars) and after decompression (yellow bars).

**Figure 3:**
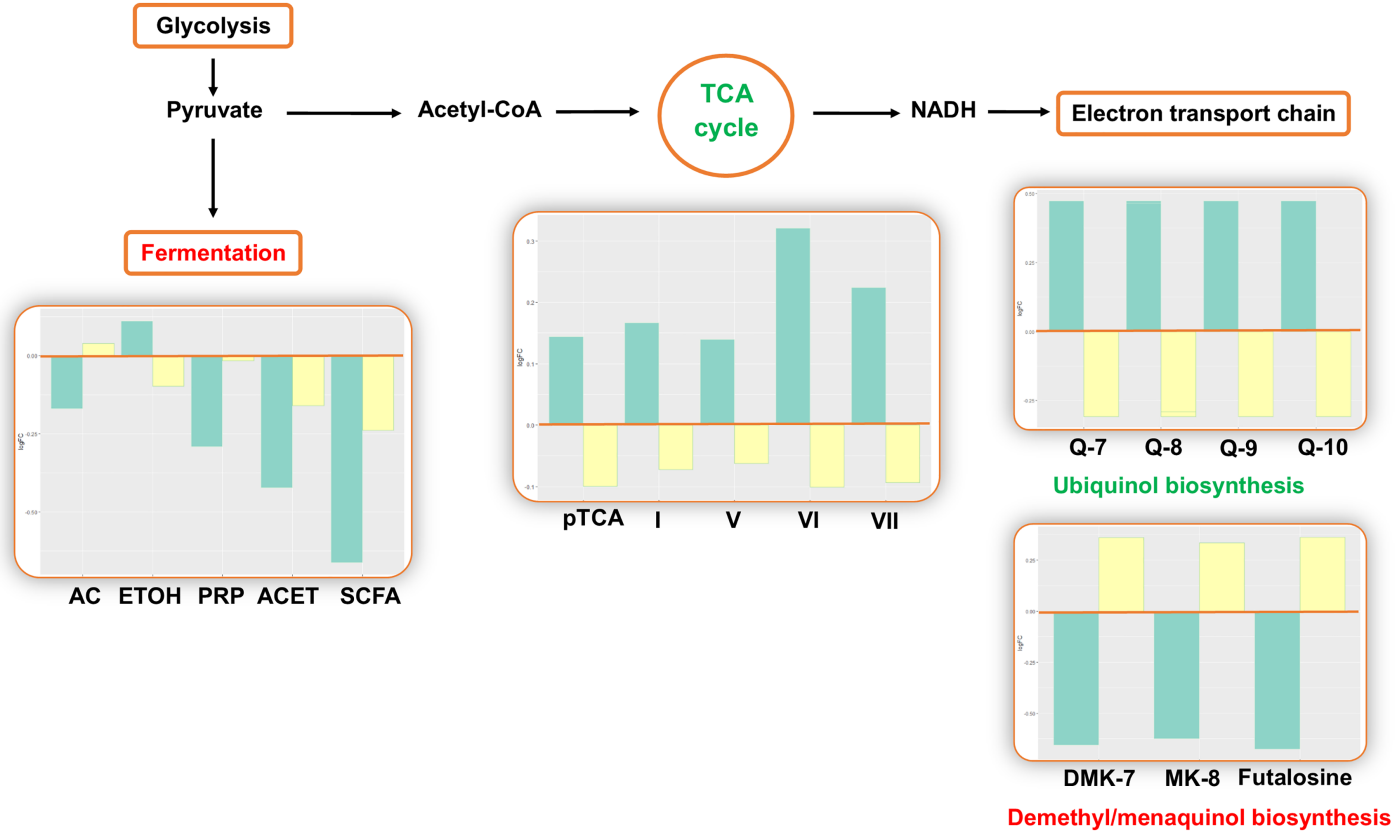
Inferred changes in bacterial energy metabolism. The bar plots show log2 fold change from paired *t* tests (means relative to baseline, *n*=23): green=after 2days of heliox saturation; yellow=after decompression. AC, acetate (*p*=0.0354); ETOH, ethanol (*p*=0.0155); PRP, propanoate (*p*=0.0234); ACET, acetone (*p*=0.0047); SCFA, short-chain fatty acids (*p*=0.0014); pTCA, partial tricarboxylic acid (TCA) cycle (obligate autotrophs, *p*=0.0240); I, TCA cycle I (prokaryotic, *p*=0.0234); V, TCA cycle V (2-oxoglutarate synthase, *p*=0.0360); VI, TCA cycle VI (Helicobacter, *p*=0.0213); VII, TCA cycle VII (acetate-producers, *p*=0.0368); Q-7, Ubiquinol-7 (*p*=0.0368); Q-8, Ubiquinol-8 (*p*=0.0368); Q-9, Ubiquinol-9 (*p*=0.0368); Q-10, Ubiquinol-10 (*p*=0.0368); DMK-7, demethylmenaquinol-7 (*p*=0.0266); and MK-8, menaquinol-8 (*p*=0.0266).

The analysis estimated changes in pathways involved in bacterial energy metabolism. There was a decrease in anaerobic pathways, including anaerobic respiration, pyruvate fermentation, and glycolysis. In aerobic pathways, the pentose phosphate pathway and tricarboxylic acid (TCA) cycle increased. In the electron transport chain of the aerobic respiration, the analysis predicted changes in electron carrier biosynthesis following the TCA cycle: The biosynthesis of ubiquinol increased, while the biosynthesis of demethyl/menaquinone decreased (Figure 3).

**Figure 4:**
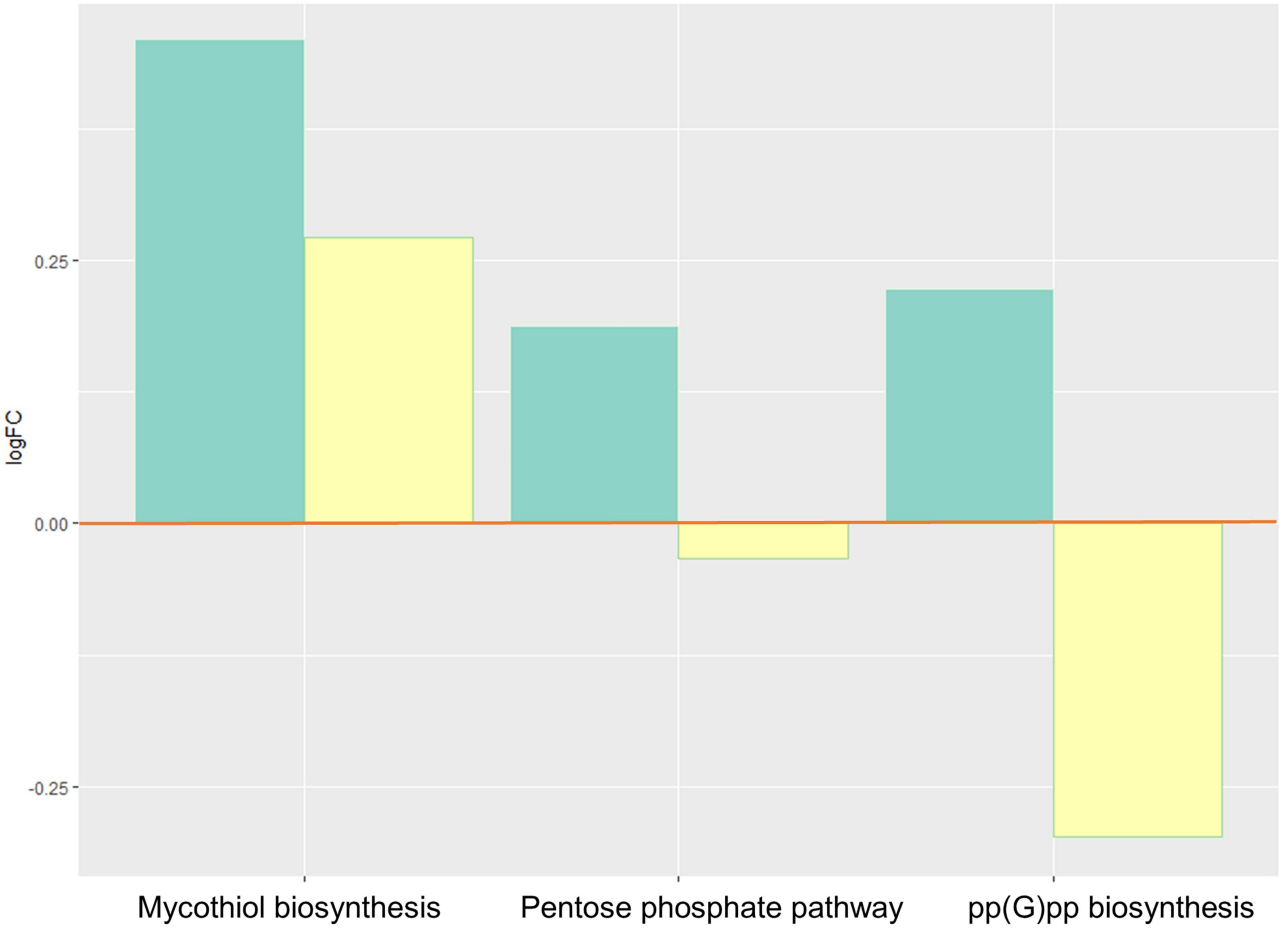
Inferred changes in bacterial oxidative stress responses: mycothiol biosynthesis (*p*=0.0274), pentose phosphate pathway (*p*=0.0047), and pp(G)pp biosynthesis (*p*=0.0429). The bar plots show log2 fold change from paired *t* tests (means relative to baseline, *n*=23): green=after 2days of heliox saturation; yellow=after decompression.

Bacterial oxidative stress responses increased (Figure 4), with elevated activity of the pentose phosphate pathway, and pp(G)pp (guanosine tetraphosphate), and mycothiol biosynthesis.

**Figure 5:**
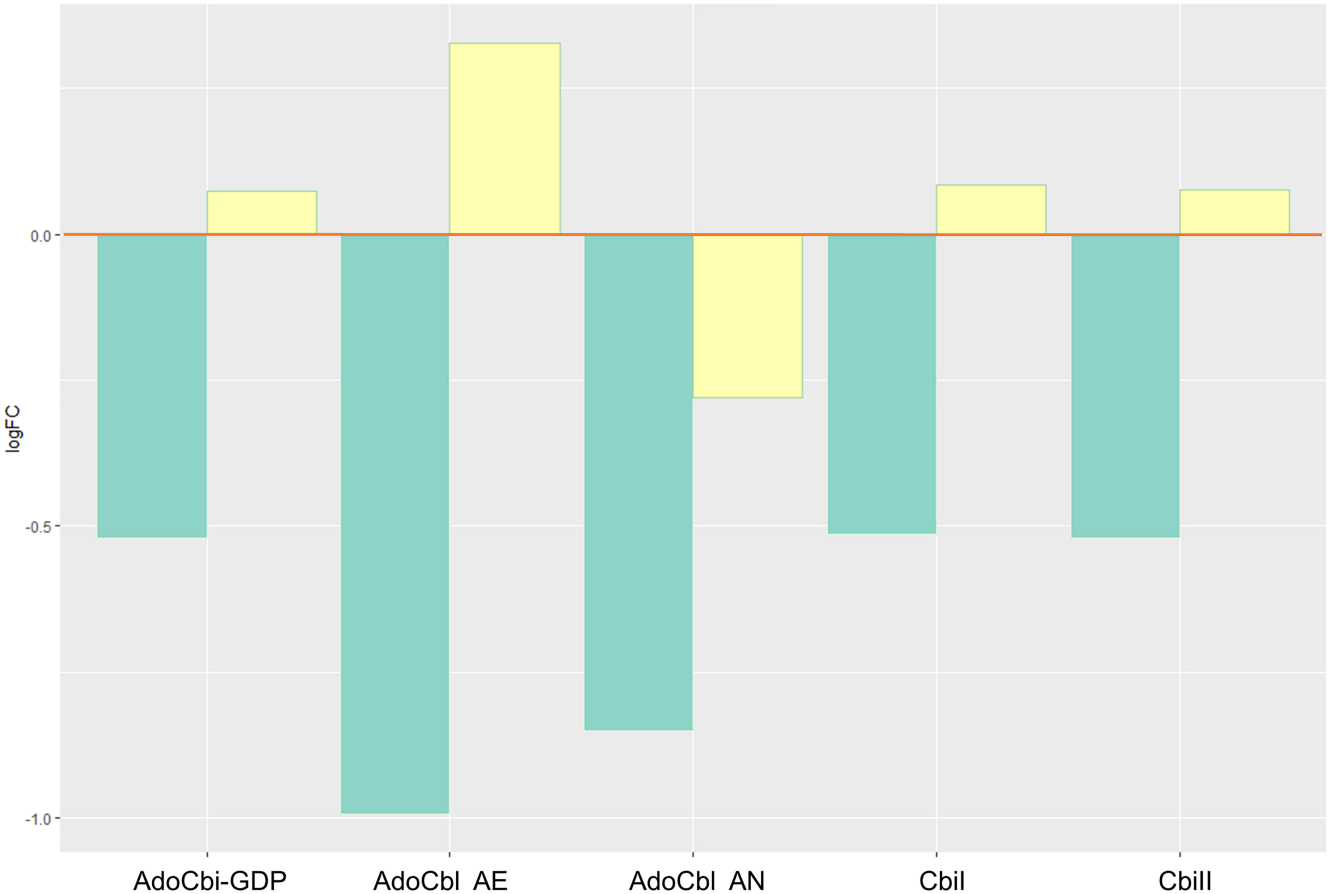
Inferred changes in bacterial vitB_12_ metabolism. The bar plots show log2 fold change from paired *t* tests (means relative to baseline, *n*=23): green=after 2days of heliox saturation; yellow=after decompression. AdoCbi-GDP, adenosylcobalamin biosynthesis from adenosylcobinamide-GDP I (*p*=0.0011); AdoCbl AE, adenosylcobalamin biosynthesis II (aerobic, *p*=0.0488); AdoCbl AN, adenosylcobalamin biosynthesis I (anaerobic, *p*=0.0338); CbiI, adenosylcobalamin salvage from cobinamide I (*p*=0.0011); and CbiII, adenosylcobalamin salvage from cobinamide II (*p*=0.0011).

Biosynthesis of bioactive vitamin B_12_ (vitB_12_) decreased through changes of the adenosylcobalamin biosynthesis pathways I (aerobic) and II (anaerobic), including adenosylcobinamide-GDP, a compound that can be used to synthesize different forms of the vitamin (Figure 5). There was also a decrease in the cobinamide salvage pathway.

The analysis also predicted changes in the biosynthesis and breakdown of universally present biomolecules, including a rise in lipid biosynthesis and fall in fatty acid biosynthesis. Furthermore, changes in the metabolism of some amino acids were predicted, with an increase in l-arginine biosynthesis and l-tyrosine degradation, and a decreased l-tryptophane degradation. Nitrogen compound metabolism was represented by a decrease in urea cycle activity (Figure 2). The other pathways that changed during the bottom phase included nucleoside and nucleotide metabolism, carbohydrate metabolism, and aromatic compound degradation (see Supplementary Data).

## Discussion

This study applied an *in silico* approach to predict changes in oral bacterial metabolism during commercial saturation diving. In light of emerging awareness of the intimate interactions between the human body and its microbiota, the results may contribute to a better understanding of physiological responses to the extreme environments in saturation diving. We used PICRUSt2 for functional abundance analysis on 16S rRNA sequence data from a previous study (Monnoyer et al., 2021) and confirmed our hypothesis of a relative increase in the abundance of aerobic pathways and a concomitant decrease in anaerobic pathways. Interestingly, the analysis also predicted decreases in several components of vitB_12_ biosynthesis.

The predicted changes in oxygen-dependent metabolism are consistent with the results from our prior taxonomic analysis, where the composition of bacterial communities was found to shift during the bottom phase in favor of aerobes (*Proteobacteria* and *Actinobacteria*) over anaerobes (*Fusobacteria*) (Monnoyer et al., 2021). An increase in pathways involved in the TCA cycle and electron carrier biosynthesis is consistent with communities in which aerobes predominate. On the contrary, the decrease in pathways of metabolites resulting from the fermentation of pyruvate may represent the decrease in relative abundance of anaerobes. There was a predicted increase in ubiquinol biosynthesis and a decrease in menaquinol and demethylmenaquinol biosynthesis. Ubiquinol, menaquinol, and demethylmenaquinol are the reduced forms of ubiquinone (coenzyme Q), menaquinone (vitamin K), and demethylmenaquinone, respectively. Ubiquinone, menaquinone, and demethylmenaquinone are all quinones (Aussel et al., 2014), which are essential components of the electron transfer chain in respiratory processes (Pandya and King, 1966; Unden, 1988). Quinones differ by their redox potential: Ubiquinone has high midpoint potential, and the midpoint potentials of menaquinone and demethylmenaquinone are low. This result is in agreement with our prior report of shifts in the bacterial composition: Whereas aerobic bacteria use ubiquinone, anaerobes use menaquinone or demethylmenaquinone (Aussel et al., 2014).

We observed an increase during hyperbaric heliox saturation of certain defense mechanisms against oxidative stress involved in bacterial survival: ppGpp biosynthesis, pentose phosphate pathway, and mycothiol biosynthesis. ppGpp (guanosine tetraphosphate) is a stress signal molecule that is usually produced under conditions of nutrient starvation (Fritsch et al., 2020). Thiols play a major role in the detoxification of stress-inducing factors, one of these thiols being glutathione (GSH). The role of the pentose phosphate pathway aside from its involvement in energy metabolism is to provide NADPH which is a cofactor of GSH reductase. ppGpp and reduced GSH are essential for maintaining the cell’s redox state and fighting oxidative stress and reactive oxygen derivatives. Diving is associated with oxidative stress from excess production of reactive oxygen species (ROS) due to hyperoxia exposure (Brubakk et al., 2014). Excess ROS promotes inflammation and can cause direct damage to cells and tissues, specifically proteins, lipids, and nucleic acids. Cells that are exposed to oxygen have evolved defensive mechanisms to limit damage from oxidative stress, and it has been shown that saturation diving causes upregulation of endogenous antioxidant defenses in human cells (Kiboub et al., 2018b). Bacteria that can use oxygen for respiration, both aerobes and facultative anaerobes, also contain a highly regulated complex of antioxidant defense enzymes. These include catalase or superoxide dismutase, as well as other non-enzymatic detoxifying mechanisms such as alkyl hydroperoxide reductase and the GSH-cycling system to overcome the toxic effects of ROS (Brioukhanov and Netrusov, 2007; Henry et al., 2014).

While the changes discussed above are reasonable in light of an oxygen-driven shift in the oral microbiota, we have no information that suggests that they affect the divers’ health and fitness. But the analyses also predicted changes in pathways associated with adenosylcobalamin biosynthesis. Adenosylcobalamin, known as coenzyme B_12_, is an active form of vitB_12_. Adenosylcobalamin is synthesized *via* the *de novo* or salvage pathways. Microbial *de novo* biosynthesis of vitB_12_ happens through two different routes: one aerobic and one anaerobic. In our analysis, both aerobic or anaerobic routes were predicted to change.

B vitamins are essential in several metabolic pathways. In saturation diving, the role of vitB_12_ in erythropoietin activity and the production of red blood cells (erythrocytes) is particularly interesting, as a decrease in biosynthesis and salvage of adenosylcobalamin in the oral microbiota may have an impact on the bioavailability of vitB_12_. Vitamin B_12_ deficiency can occur through malabsorption, dietary insufficiency, and autoimmune conditions (e.g., pernicious anemia) (Ankar and Kumar, 2021). Mild anemia is common after saturation diving since hyperbaric environment causes a depression of erythropoiesis (Kiboub et al., 2018a) and hemoglobin (Luczynski et al., 2019). Anemia is hypothesized to be partly responsible for the fatigue that saturation divers experience up to 1week after decompression (Imbert et al., 2018), and vitB_12_ is among the nutritional factors that should be monitored (Deb et al., 2016). Correlations have been observed between nutrient intake (e.g., vitamins) and its impact on the microbial abundance, diversity, and richness of the oral microbiome (Kato et al., 2017). Moreover, data from gut microbiota indicate that bacteria contribute to the supply of vitB (vitB_12_ included) to their human hosts (Magnúsdóttir et al., 2015). Daily requirements for vitB_12_ are typically achieved through a diverse diet containing meat, fish, and fortified foods; however, in some cases, supplementation may be required, if availability is insufficient through food intake. Our findings are consistent with recommendations for vitB_12_ supplements as part of the divers’ diet.

### Limitations

The analytic approach in this study comes with some caveats. First, as the PICRUSt2 software uses metagenomic data for functional predictions, the results are a direct reflection of the relative abundance of the bacteria that harbor these functions. By extension, the analysis does not determine changes in metabolic activity within individual bacteria. Second, this analysis can only identify functions in bacteria for which metagenomic data were present in the reference. To this second point, our analysis predicted changes in the metabolism of cellular biomolecules that are universally present in bacteria: nucleosides, nucleotides, lipids, amino acids, and carbohydrates. These results may be artifacts caused by the absence of data from bacteria for which 16S rRNA sequences were not annotated in KEGG at the time of the analysis (Langille, 2018). Therefore, the conclusions must be tempered to whether they are reasonably linked by biology to the environmental stressors in saturation diving and whether they are likely to be of consequence for the divers’ health and fitness. To confirm our findings, future studies should include direct measurements of metabolites.

## Conclusion

This study is the first to examine the metabolic activity of oral microbiota during commercial saturation diving. Whereas the predicted changes were transient and appeared primarily to be associated with the survival and growth of bacteria in hyperoxic environments, a reduction in bacterial vitB_12_ biosynthesis during the bottom phase may hypothetically disturb the divers’ physiology. While there is limited knowledge of how oral bacterial metabolism affects human physiology, the results from this study are in line with published recommendations for vitB_12_ supplements as part of the divers’ diet.

## Data Availability Statement

The original contributions presented in the study are included in the article/Supplementary Material, further inquiries can be directed to the corresponding author.

## Ethics Statement

The studies involving human participants were reviewed and approved by Norwegian Regional Committee for Medical and Health Research Ethics (REK). The patients/participants provided their written informed consent to participate in this study.

## Author Contributions

RM, IE, and JL designed the study. IE collected the material. RM, JL, and AH conducted the analyses. RM, IE, SD, KH, and JL contributed to the interpretation of the results. All authors contributed to the article and approved the submitted version.

## Funding

This study was part of a Knowledge-Building Projects for Industry, placed at NTNU, Norway and funded from the Norwegian Research Council and Equinor on behalf of PRSI Pool through the Large-Scale Programme for Petroleum Research (PETROMAKS2: project no. 280425) *via* an integral part dedicated to research on Health, Safety, and Environment (HSE) in the petroleum sector.

## Conflict of Interest

TechnipFMC sponsored helicopter transfers and boarding on the DSV Deep Arctic for IE.

The remaining authors declare that the research was conducted in the absence of any commercial or financial relationships that could be construed as a potential conflict of interest.

## Publisher’s Note

All claims expressed in this article are solely those of the authors and do not necessarily represent those of their affiliated organizations, or those of the publisher, the editors and the reviewers. Any product that may be evaluated in this article, or claim that may be made by its manufacturer, is not guaranteed or endorsed by the publisher.

## Footnotes

^1^ https://www.ebi.ac.uk/ena/browser/view/PRJEB40804
